# Serotonin 2a Receptor and Serotonin 1a Receptor Interact Within the Medial Prefrontal Cortex During Recognition Memory in Mice

**DOI:** 10.3389/fphar.2015.00298

**Published:** 2015-12-23

**Authors:** Juan F. Morici, Lucia Ciccia, Gaël Malleret, Jay A. Gingrich, Pedro Bekinschtein, Noelia V. Weisstaub

**Affiliations:** ^1^Systems Neuroscience Group, Laboratory of Experimental Cognition and Behavior, Institute of Physiology and Biophysics, IFIBIO “Houssay,” CONICET and University of Buenos Aires Medical SchoolBuenos Aires, Argentina; ^2^Lyon Neuroscience Research Center, Centre National de la Recherche Scientifique UMR 5292 – Institut National de la Santé et de la Recherche Médicale U1028 - Université Claude Bernard Lyon1Lyon, France; ^3^Sackler Institute for Developmental Psychobiology, Columbia University, New YorkNY, USA; ^4^New York State Psychiatric InstituteNew York, NY, USA; ^5^Laboratory of Memory Research and Molecular Cognition, Institute for Cell Biology and Neuroscience, CONICET and University of Buenos Aires Medical SchoolBuenos Aires, Argentina

**Keywords:** serotonin, 5-HT2A receptor, 5-HT1A receptor, recognition memory, interference control

## Abstract

Episodic memory, can be defined as the memory for unique events. The serotonergic system one of the main neuromodulatory systems in the brain appears to play a role in it. The serotonin 2a receptor (5-HT2aR) one of the principal post-synaptic receptors for 5-HT in the brain, is involved in neuropsychiatric and neurological disorders associated with memory deficits. Recognition memory can be defined as the ability to recognize if a particular event or item was previously encountered and is thus considered, under certain conditions, a form of episodic memory. As human data suggest that a constitutively decrease of 5-HT2A signaling might affect episodic memory performance we decided to compare the performance of mice with disrupted 5-HT2aR signaling (*htr2a*^−/−^) with wild type (*htr2a*^+/+^) littermates in different recognition memory and working memory tasks that differed in the level of proactive interference. We found that ablation of 5-HT2aR signaling throughout development produces a deficit in tasks that cannot be solved by single item strategy suggesting that 5-HT2aR signaling is involved in interference resolution. We also found that in the absence of 5-HT2aR signaling serotonin has a deleterious effect on recognition memory retrieval through the activation of 5-HT1aR in the medial prefrontal cortex.

## Introduction

Serotonin (5-HT) is synthesized in neurons of the raphe nuclei localized in the brain stem. These cells project their heavily ramified axons throughout the brain (Jacobs and Azmitia, 1992). 5-HT exerts its multiple functions through 7 distinct families of receptors (Humphrey et al., 1993; Hoyer et al., 1994; Hoyer and Martin, 1996). Each family is composed by several members that differ in localization and downstream signaling (Hoyer et al., 2002; Seyedabadi et al., 2014). The serotonin 2a receptor (5-HT2aR), one of the principal post-synaptic receptors for 5-HT, is localized in the cortex, ventral striatum, hippocampus, and amygdala (Pompeiano et al., 1994; Cornea-Hébert et al., 1999; López-Giménez et al., 2001), brain structures involved in memory processes. As many other 5-HT receptors, the 5-HT2aR is a G-coupled protein receptor. It has a complex signaling mechanisms including activation of Gq pathway, and scaffolding proteins, including Beta-arrestin 2 (Berg et al., 1998; Schmid et al., 2008; Schmid and Bohn, 2010). 5-HT2aR is expressed in excitatory and inhibitory cells. It has a very characteristic laminar distribution in all cortical sub regions with a dorsal ventral gradient (Jakab and Goldman-Rakic, 1998). The distribution of 5-HT2aR –highly expressed in the apical dendrites of pyramidal neurons in layer 5 of the cortex- suggests that cortical 5-HT2aR modulate cortical function via distinctive mechanisms (Jakab and Goldman-Rakic, 1998) and thus play a key role in the modulation of different cortical functions. Interestingly, the serotonin 1a receptor (5-HT1aR), a Gi coupled receptor, and 5-HT2aRs appear to be co-expressed in a large fraction of pyramidal cells (Araneda and Andrade, 1991; Amargos-Bosch et al., 2004; Béïque et al., 2004) in the medial Prefrontal Cortex (mPFC). Therefore, they may regulate in a cooperative manner the way pyramidal neurons encode excitatory inputs into action potential firing. However, how this interaction affects behavior is still unclear.

Episodic memory can be defined as the memory for unique events that have as a characteristic, particular temporal and spatial features that allows an experience to be considered as a sole event. This type of memory is fundamental for an individual to construct his/her own autobiographical memory (Tulving, 1984; Schacter et al., 2011). From human and animal studies we have gained information about the brain structures, mechanisms underlying this type of memory (Schott et al., 2006a,b; Seyedabadi et al., 2014) and it has been shown that the serotoninergic system plays a particular role on it (Meneses, 1999, 2015; de Quervain et al., 2003; Meneses et al., 2004, 2011; Meneses and Liy-Salmeron, 2012; Seyedabadi et al., 2014). In healthy individuals, 5-HT2aR might be involved in memory performance (de Quervain et al., 2003; Sigmund et al., 2008) and a common polymorphism at position 452 (His to Tyr) was associated with decrease episodic memory (de Quervain et al., 2003; Sigmund et al., 2008; Avgan et al., 2014). Also 5-HT2aR have been has been implicated in different neuropsychiatric and neurological disorders including schizophrenia, attention deficit hyperactive disorder, and Alzheimer's disease (Meltzer et al., 2003; Norton and Owen, 2005; Mestre et al., 2013; Selvaraj et al., 2014). All of them are associated with memory deficits.

Recognition memory can be defined as the ability to recognize if a particular event or item was previously encountered and is thus considered, under certain conditions, a form of episodic memory (Morici et al., 2015). In animal models, recognition memory can be evaluated using a spontaneous novel object recognition task (SNOR). This task and all its variants exploit the natural tendency of rodents to explore novel stimuli over familiar stimuli. A major advantage of these tasks is the fact that they are based on the natural preference of an animal to explore novel objects and are simple, and less stressful or time consuming than other traditional memory tasks. Using these tasks, we have previously showed that the blockade of 5-HT2aR in the mPFC before a test session affects the performance of rats in recognition tasks that cannot be solved by a single item strategy (Bekinschtein et al., 2013).

Memories are not isolated in the brain. Different experiences are often associated to the same cues which could diminish correct access to a given memory during retrieval. In this way, the memories for different experiences can compete during retrieval causing interference. Experiments in humans have suggested that the PFC participates in retrieval control and selection of the relevant memory traces (Squire et al., 2004; Ferbinteanu et al., 2006). Our results in animal studies allowed us to propose that 5-HT2aR signaling in the mPFC is involved in the ability of this structure to control memory interference during retrieval when retrieval cues are not unambiguously linked to a specific memory trace. Interestingly the same result was observed when 5-HT1AR are activated suggesting that the serotoninergic modulation of the mPFC during the retrieval of recognition memory task involves opposite effects through these two different receptors (Bekinschtein et al., 2013).

Because human data suggest that a constitutively decrease of 5-HT2A signaling might affect episodic memory performance (de Quervain et al., 2003; Sigmund et al., 2008), we decided to study recognition memory in a model that constitutively lacks 5-HT2aR activity. We compared the performance of mice with disrupted 5-HT2aR signaling (*htr2a*^−/−^) with wild type (*htr2a*^+/+^) littermates in recognition memory tasks. We also compared the performance of these mice in two working memory tasks that differed in the level of proactive interference. In order to understand the interaction within the serotoninergic system during the modulation of episodic memory, we also analyzed the role of 5HT1aR.

## Materials and methods

### Experimental animals

Generation of genetically modified *htr2a*^−/−^ mice and their control (*htr2a*^+/+^) littermates was described elsewhere (Weisstaub et al., 2006). Animals were housed at 12 h light/dark cycle at 23°C with food and water *ad libitum*. Experiments took place during the light phase of the cycle (between 10 a.m. and 5 p.m., see exception below) in quiet room with dim light. The experimental protocol for this study was approved by the National Animal Care and Use Committee of the University of Buenos Aires (CICUAL). All experiments were performed on adult (8–16 weeks old) male mice. Eight to ten animals per genotype were used for each experiment.

### Apparatus and behavioral experiments

Spontaneous novel object recognition and temporal memory object recognition tasks were conducted in a rectangular shaped apparatus. Briefly, the rectangular arena had homogenous gray walls constructed from opaque Plexiglas. The apparatus was 40 × 25 cm length × 30 cm high. For object in context task, an additional apparatus was used. It was a triangular arena made of homogenous walls constructed from opaque gray Plexiglas. It was 40 × 25 cm length × 30 cm high. Both contexts had the same surface area in order to avoid differences due to the size of the arena. Duplicate copies of objects made from plastic, glass and aluminum were used. The height of the objects ranged from 8 to 12 cm and they varied with respect to their visual and tactile qualities. All objects were affixed to the floor of the apparatus with an odorless reusable adhesive to prevent them for being displaced during each session. The objects were always located along the central line of the maze, away from the walls and equidistant from each other. As far as we could determine the objects had no natural relevance for the mice as they were never associated to any reinforcement. The objects, floor and walls were cleaned with ethanol 10% between experiments.

The Y-maze spontaneous alternation test was conducted in a maze with three identical arms of transparent Plexiglas (40 × 4.5 × 12 cm). Visual cues were located in the periphery of the room to allow spatio-visual orientation.

The radial arm maze (RAM) test was conducted in a radial 8-arm maze described elsewhere (Saxe et al., 2007). The apparatus consisted in an octagonal central platform connected to eight arms. From this platform, doors made of Plexiglas could be automatically lowered by the experimenter in order to allow the entry of the animals into the arms of the maze.

#### Spontaneous novel object recognition task (SNOR)

To address whether simple object recognition memory was affected by the constitutive lack of 5-HT2aR, we used a SNOR task. Each trial consisted of three phases (see **Figure 2A**). During habituation sessions, animals were introduced into the arena for 10 min during the first session. In the subsequent habituation sessions the mice were exposed for 5 min each time. During the sample phase, two identical objects (A1 and A2) were placed into the arena. The mice were re-introduced into the arena facing the wall (and not the objects). They were then allowed to explore the objects during 10 min. The time spent exploring the two objects were scored by an experimenter observing the mouse from a distance. Exploration of an object was defined as directing the nose to the object at a distance of <2 cm and/or touching it with the nose. Turning around or sitting on the object was not considered exploratory behavior. Mice that explored less than 5 s were excluded from the experiments.

At the end of the sample phase, the mouse was removed from the apparatus and returned to its home cage for the duration of the retention period of 24 or 3 h. After this delay, the mouse was placed back into the apparatus for the test session. In this case, the arena now contained an identical copy of the sample (familiar) object (A3) and a new object (B). The position (left or right) in which the objects were placed was counterbalanced between animals. The mouse was allowed to explore the objects for a period of 5 min, at the end of which it was removed and returned to its home cage. We calculated a discrimination index (DI) defined as the proportion of total exploration time spent exploring the novel object (i.e., the difference in time spent exploring the novel and familiar objects divided by the total time spent exploring the objects).

#### Object in context recognition task (OIC)

**Figure 1:**
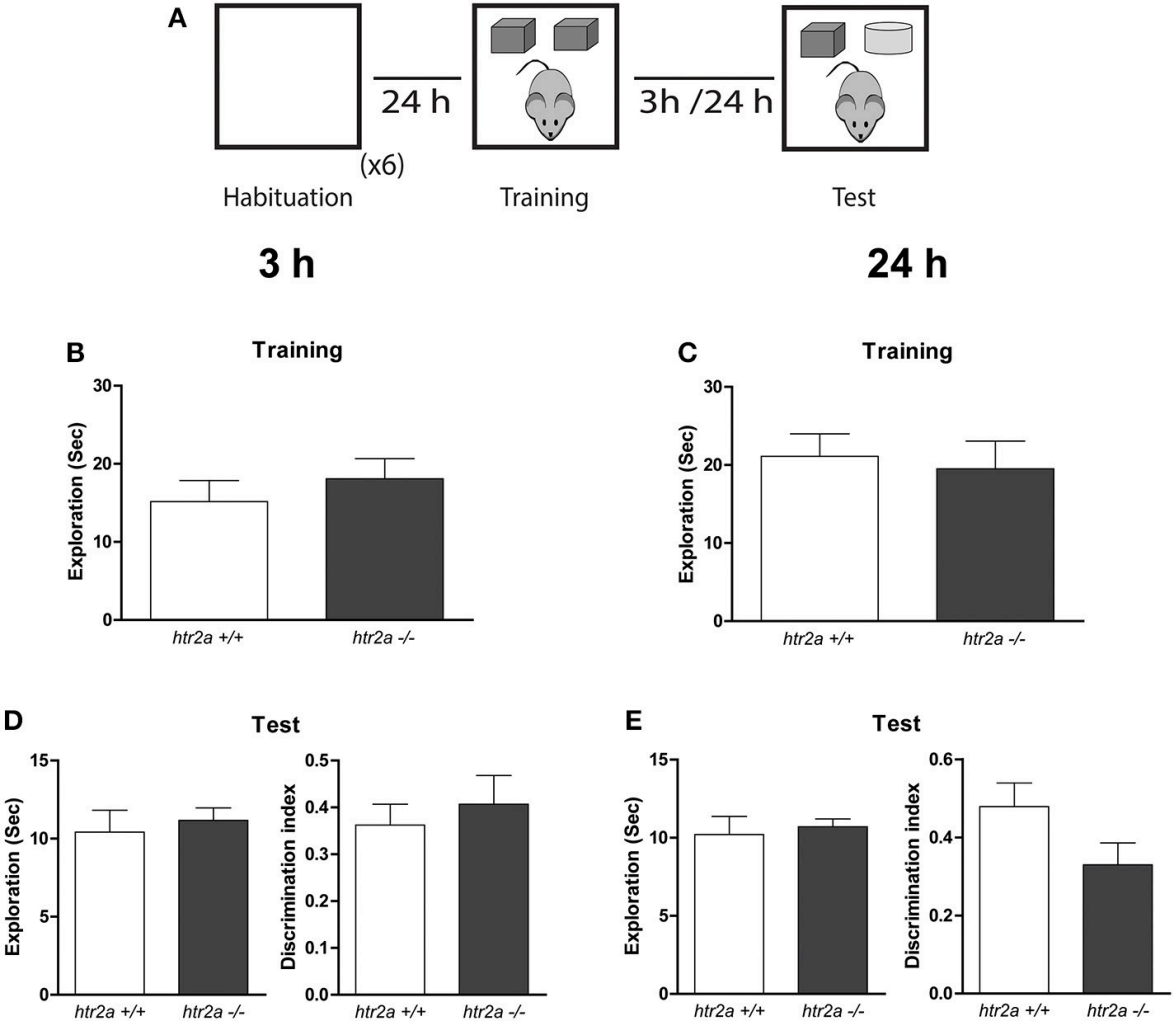
**5-HT2aR is not required for the single-item object recognition**. **(A)** Training and Testing scheme. The mice were exposed to a context containing two identical copies of an object for 10 min. Three or twenty-four hours later they were re-exposed for 5 min to the same context of the training phases containing one copy of the objects previously presented and a copy of a new object. **(B,C)** Exploration time measured in seconds made by the mice during the training. **(D,E)** Total exploration measured in seconds (left) and Discrimination index (right) obtained from the test phase delayed 3 or 24 h from the training phase. DI was calculated as the time spent exploring the novel object minus the time spent exploring the familiar object over the total exploration time. *n* = 9–11 per group, *p* > 0.05, Student's *t*-test.

In order to evaluate if the absence of 5-HT2a signaling was involved in other recognition tasks, we used the OIC task. The habituation phase was similar to the one used in SNOR, but in this case, the mice were habituated to two different contexts, 10 min in each context. On sample phase 1, subjects were placed in context 1 facing the wall opposite to the objects and were allowed to explore two identical objects (A1 and A2) for 10 min (see Figure 1A). In sample phase 2, conducted 1 h later, mice were placed in context 2 together with two identical new objects (B1 and B2) and were allowed to explore the objects for 10 min. The objects used had the same characteristics described in the previous experiments. Memory was tested 24 h later. On the test phase, mice were reintroduced to context 1 or 2 (pseudo randomly assigned) and were allowed to explore freely for 5 min one copy of object A and one copy of object B. The time spent exploring the two objects were scored during the testing phase. We calculated a discrimination index defined as the proportion of total exploration time spent exploring the object not previously associated to a given context (i.e., the difference in time spent exploring the object not previously associated to a given context ant the familiar object divided by the total time spent exploring both objects).

#### Temporal order recognition task (TMOR)

*To address if recency memory was affected by the constitutive* blockade of 5-HT2aR expression, we conducted a TMOR task. This task comprised one 10 min habituation session, two sample phases and one test trial (see **Figure 3A**). It was conducted in the same arena used for the SNOR or in any of the arenas used for the Object in Context Task (OIC; see next paragraph). The habituation phase was similar to the one used in the SNOR task described above. During two sample phases, the subjects were allowed to explore two identical copies of an object for 10 min. Different objects were used for sample phases 1 and 2, with a delay between the sample phases of 1 h. The test trial (5 min duration) was given 3 h after sample phase 2. During the test trial, a copy of the objects from sample phase 1 and a copy of the objects from sample phase 2 were used. The positions of the objects in the test phase and the objects used in sample phase 1 and sample phase 2 were counterbalanced between the animals. We calculated a discrimination index defined as the proportion of total exploration time spent exploring the less recently presented object (i.e., the difference in time spent exploring the less recently presented object and the more recently presented object divided by the total time spent exploring both objects).

#### Radial 8-arm maze test

Food-deprived mice (85% of *ad-libitum* weight) were habituated for 10 days to retrieved food pellets at the end of the eight arms. The mice used distal visual cues located in the walls surrounding the maze for spatial orientation. After habituation sessions, mice were placed on the central orthogonal platform. In order to reduce inter-trial interference, subjects performed one trial per day, consisting of a sample phase and a test phase. During the sample phase, animals were allowed to explore only four (pseudo-randomly pre-dertermined) arms. After exploring these four arms, the experimenter closed the doors of these arms. During this sample phase, re-entering the previously visited arm was considered as an error. The test phase was then conducted 5 s after the sample phase had ended. During this test phase, all arms were opened but only the previously locked (and therefore not yet visited) arms contained food. The exploration of a previously visited arm (during sample phase), was considered as an error. Animals were exposed to one trial per day during 10 days.

#### Y-maze spontaneous alternation test

*The Y-shaped maze consisted of three identical* arms of transparent Plexiglas (43 × 4 × 12.5 cm) placed at 120° angles to each other (Belforte et al., 2010; Braz et al., 2015). Mice were placed at the end of one arm facing the center and allowed to explore the maze freely for 8 min without training, reward, or punishment. All sessions were video recorded through a camera mounted above the maze allowing to analyze behavior of the mice by scoring the videos offline. Entries into each arm were scored and alternation behavior was defined as a complete cycle of consecutive entrances into each of the 3 arms without repetition. The percentage of spontaneous alternation was calculated as the number of alternations divided by the possible alternations [(# alternations)/(total arm entries − 2)]. Total entries were scored as an index of ambulatory activity in the Y maze and mice with scores below 7 were excluded as they showed a very low level of exploration. All experiments were conducted during the initial dark phase (6:00 p.m. to 9:00 p.m.) to maximize exploratory behavior to consistently obtained high number of entries (Belforte et al., 2010).

### Surgery and drug infusions

The mice were deeply anesthetized with ketamine (150 mg/kg) and xilacine (6.60 mg/kg) and placed in a stereotaxic frame. The skull was exposed and adjusted to place bregma and lambda on the same horizontal plane. Small burr holes were then drilled and a set of 23 G guide cannulae of 0.5 cm were implanted bilaterally into the mPFC [anterior-posterior (AP) +1.5 mm; lateral(L) ±0.5 mm; dorsoventral (DV) −0.80 mm]. Cannulae were fixed to the skull with dental acrylic. At the end of surgery, animals were injected with a single dose of meloxicam (0.33 mg/kg) as analgesic and gentamicine (5 mg/kg) as antibiotic. Behavioral procedures commenced 5–7 days after surgery. We used a within subject design, each animal was evaluated twice, once with vehicle and once with the drug. Half of the animals were injected first with vehicle and half fist with the drug. Mice from each genotype received infusion of VEH and WAY-100135 separated by 7 days. The order of infusions was randomly assigned. On the test day, infusions were made using a 30 G injection cannula connected to a 10 μl Hamilton syringe. Cannulated mice received bilateral 0.5 μl infusions of WAY-100135 (5-HT1aR antagonist) or DMSO 13% into the mPFC 15 min before the test session. WAY-100135 was diluted in DMSO 13% into final concentration of 2μg/μl (Carli et al., 1995).

### Statistical analysis

Data were expressed as mean ± SEM and analyzed with Student's *t*-test, One-way analyses of variance (ANOVAs); Two-way ANOVA with and without repeated measures were also used when required. Factors were: Genotype for the One-way ANOVA and Genotype and Treatment for the Two-way ANOVA analyses were followed by *post-hoc* tests. Statistical analyses were performed using Graph Pad Prism 5. *P* < 0.05 was considered significant.

## Results

### *Htr2a*^−/−^ response is normal in the SNOR task

To study whether 5-HT2aR deficiency caused a deficit in recognition memory, we exposed *htr2a*^+/+^ and *htr2a*^−/−^ mice to a SNOR task. This task can be solved by a single item strategy. Mice only require to recognize if the objects presented are familiar or novel. We found that the constitutive blockade of 5-HT2aR signaling has not affect on how mice distributed their exploratory time between the copies of the objects during the training phase [*htr2a*^+/+^: *t*_(8)3h_ = 1.03, *p* = 0.329; *t*_(10)24h_ = 1.41, *p* = 0.186. *htr2a*^−/−^: *t*_(7)3h_ = 0.92, *p* = 0.386; *t*_(8)24h_ = 0.677, *p* = 0.517] or the total exploratory levels [see Figures 1B,C; *t*_(15)3h_ = 0.7832, *p* = 0.7745; *t*_(18)24h_ = 0.3515, *p* = 0.7396]. Neither in the ability of the animals to discriminate between a familiar and a novel object as shown by a non-different discrimination index or total exploratory times when animals were tested 3 h. [see Figure 1D; *t*_(15)_ = 0.5949, *p* = 0.4863] or 24 h [see Figure 1E; *t*_(18)_ = 1.777, *p* = 0.6230] after training (sample phase). This result indicates that blockade of 5-HT2a signaling is not necessary for object recognition *per se* and that the *htr2a*^−/−^ mice have a normal ability to acquire and consolidate recognition memory.

### *Htr2a*^−/−^ mice showed deficits in the OIC task

**Figure 2:**
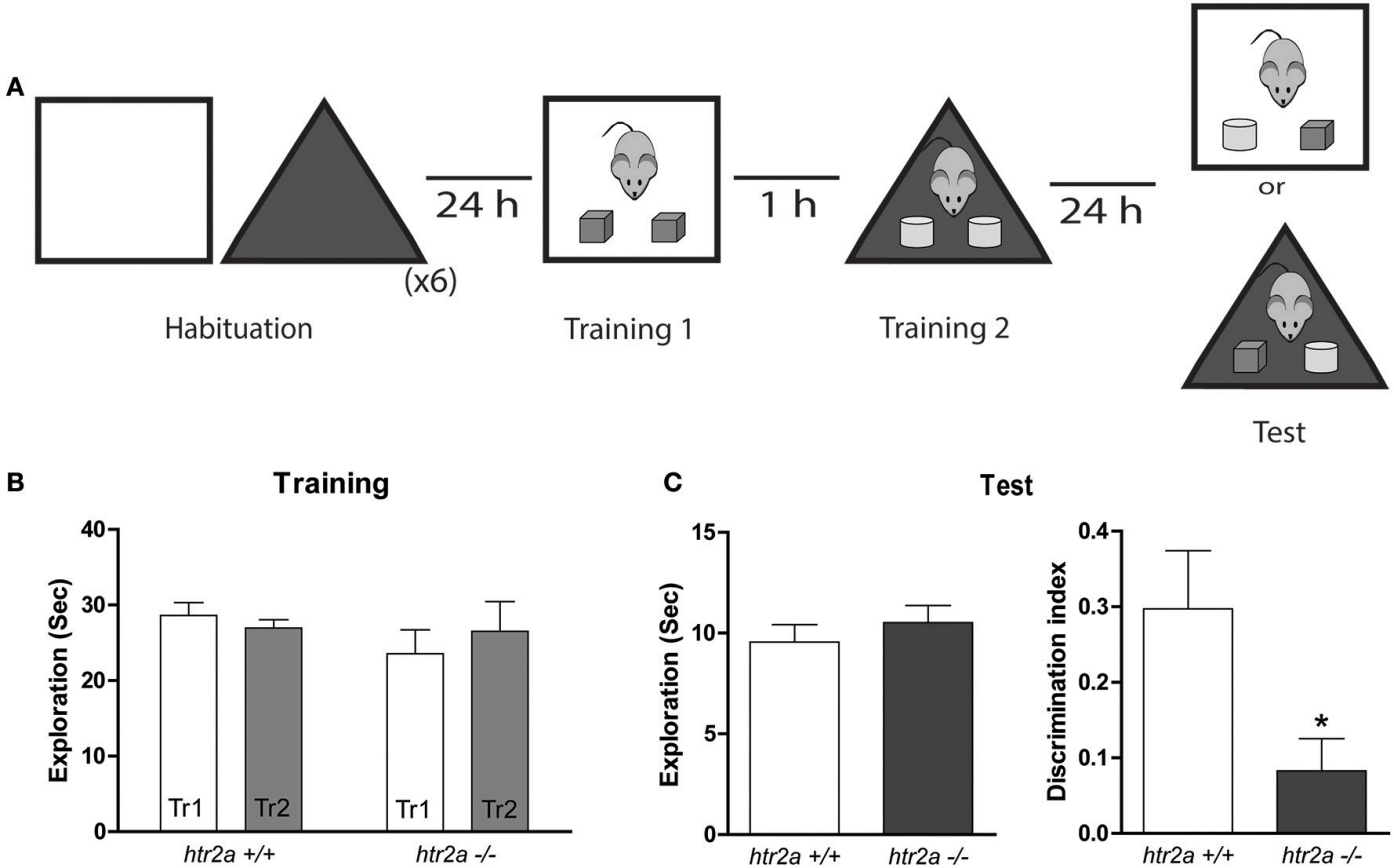
**5-HT2aR is required for the object-in-context task**. **(A)** Training and Testing scheme. Mice were exposed to a context containing two identical copies of an object. An hour later they were exposed to a different context containing two identical copies of a different object. Twenty-four hours later they were re-exposed to one of the context containing one copy of each of the objects. **(B)** Exploration time measured in seconds made by the mice during the first and the second Training phases (Tr 1 and Tr2). **(C)** Total exploration measured in seconds (left) and Discrimination Index (right). DI was calculated as the time spent exploring the incongruent object minus the time spent exploring the congruent object over the total exploration time during the test session. *n* = 10–11 per group, ^*^p < 0.05, Student's *t*-test.

The OIC is a task that specifically evaluates the ability of the animals to recognize the “what and where” features of memory and, unlike the SNOR task, it has been shown to be dependent on the integrity of the PFC (Spanswick and Dyck, 2012; Bekinschtein et al., 2013). The OIC task is a three trial procedure divided in two sample phases and one test phase (see Figure 2A). During the sample phase, two different pairs of identical objects are presented in different contexts. During the test phase, a copy of each of the objects is presented in one of the previously experienced contexts. Thus, while one of the objects is presented in the same context experienced during the training session (congruent), the other object has not been experienced in this particular context, generating a discrepancy between the object and the context (incongruent). In this task, the novelty comes from the novel combination of an object and a context, and this will drive exploration. Recognition of this novel combination will be related to the ability of the animal to remember in which context an object presented during training. This task presents a higher load of interference than the SNOR, because during test the animals experience two familiar objects and these two memory traces can compete for retrieval.

We found that *htr2a*^−/−^ mice showed a deficit in the level of discrimination of the congruent and incongruent objects as indicated by their null discrimination index during the test phase and compared with *htr2a*^+/+^ [see Figure 2C; *t*_(19)_ = 2.4998, *p* = 0.0218]. This deficit was not due to differences in the total exploratory time during the test phase [see Figure 2C; *t*_(19)_ = 0.789, *p* = 0.4397] or during the sample phases [see Figure 2B; *F*_genotype(1, 20)_ = 0.4908 Two-way ANOVA] suggesting that the deficit might arise from the inability of *htr2a*^−/−^ mice to recognize a novel combination of an object and a context. Although our model does not allow us to show which memory phase is affected by the mutation. The results obtained in the SNOR task suggest that the deficits observed in the OIC task are not due to a general deficit in acquisition, or consolidation but rather from something particular in the comparisons the animal has to make during retrieval.

### *Htr2a*^−/−^ mice showed deficits in the TMOR task

**Figure 3:**
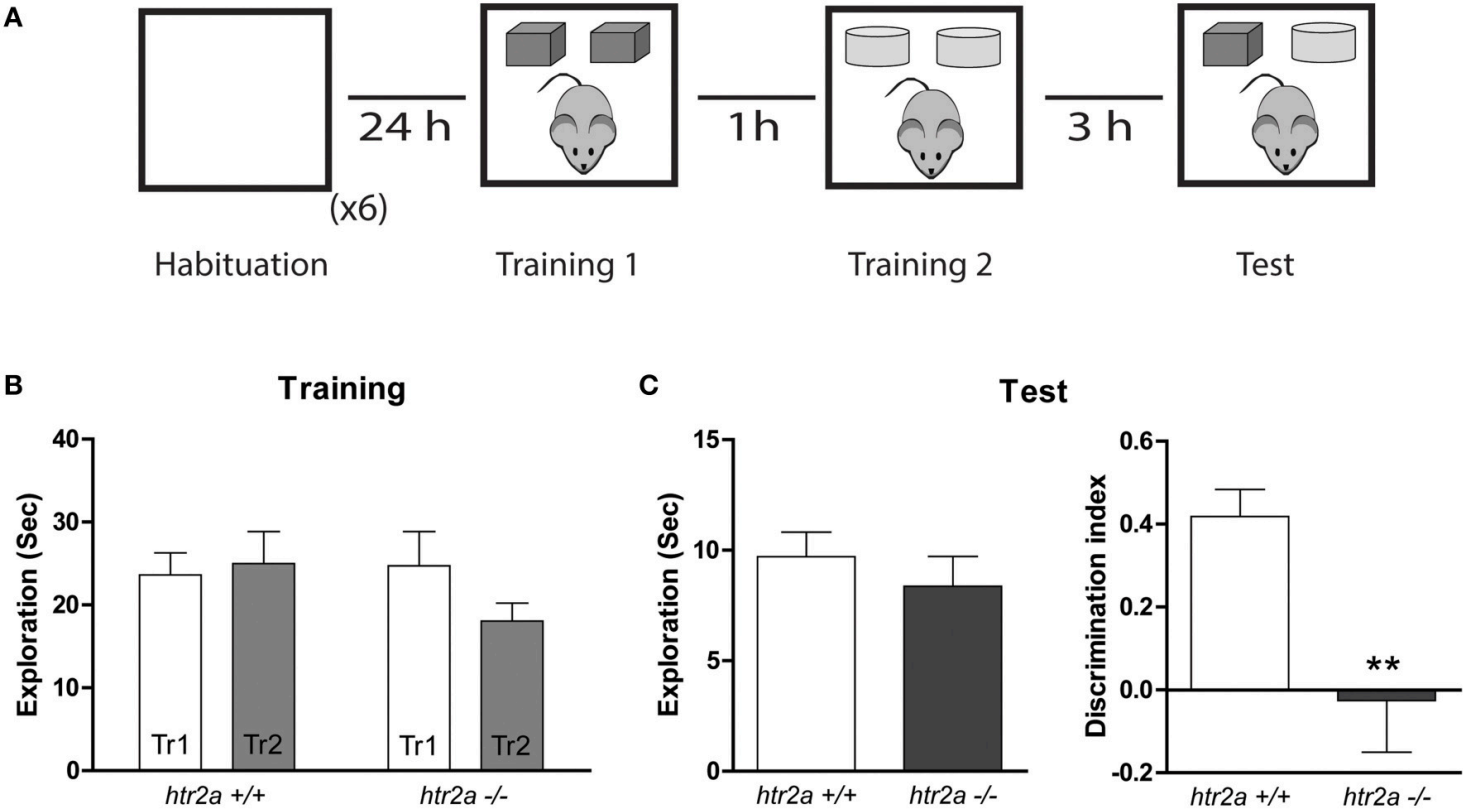
**5-HT2aR is required for the temporal order recognition task. (A)** Training and Testing scheme. The mice were exposed to an arena containing two identical copies of an object and an hour later they were re-exposed to the same arena containing two identical copies of a different object. Three hours later animals were exposed to the same context containing one copy of each object (“older” and “newer”). **(B)** Exploration time measured in seconds during the first and the second Training phases (Tr 1 and Tr2). **(C)** Total exploration measured in seconds (left) and Discrimination Index (right). DI was calculated as the time spent exploring the older object minus the time spent exploring the newer object over the total exploration time during the test session. *n* = 10 per group, ^**^p < 0.01, Student's *t*-test.

The TMOR measures the ability of the animals to assess the temporal order of two different object presentation events. The task is composed of two sample phase separated by 1 h and a retention phase performed 3 h later (see Figure 3A). In this paradigm, animals usually display a greater exploration time of the less recently presented “older” object. *Htr2a*^+/+^ and *htr2a*^−/−^ mice were trained and tested in this paradigm. There were no significant differences between genotypes in the total exploration time during the sample [see Figure 3B; *F*_genotype(1, 18)_ = 0.5307]; or test [see Figure 3C; *t*_(18)_ = 0.7843, *p* = 0.1964] phase. However, the distribution of the time exploring the objects differed between *htr2a*^+/+^ and *htr2a*^−/−^ mice. The discrimination index shows that *htr2a*^−/−^ explored both objects to the same extent showing no recency discrimination while the *htr2a*^+/+^ explored more the “older” object compared with the most “recent” one [see Figure 3C; *t*_(18)_ = 3.153, *p* = 0.0055] suggesting that 5-HT2aR signaling is necessary to be able to identify the order in which two objects were previously encountered.

### *Htr2a*^−/−^ mice showed deficits in the Y-maze task but not in the radial arm maze

**Figure 4:**
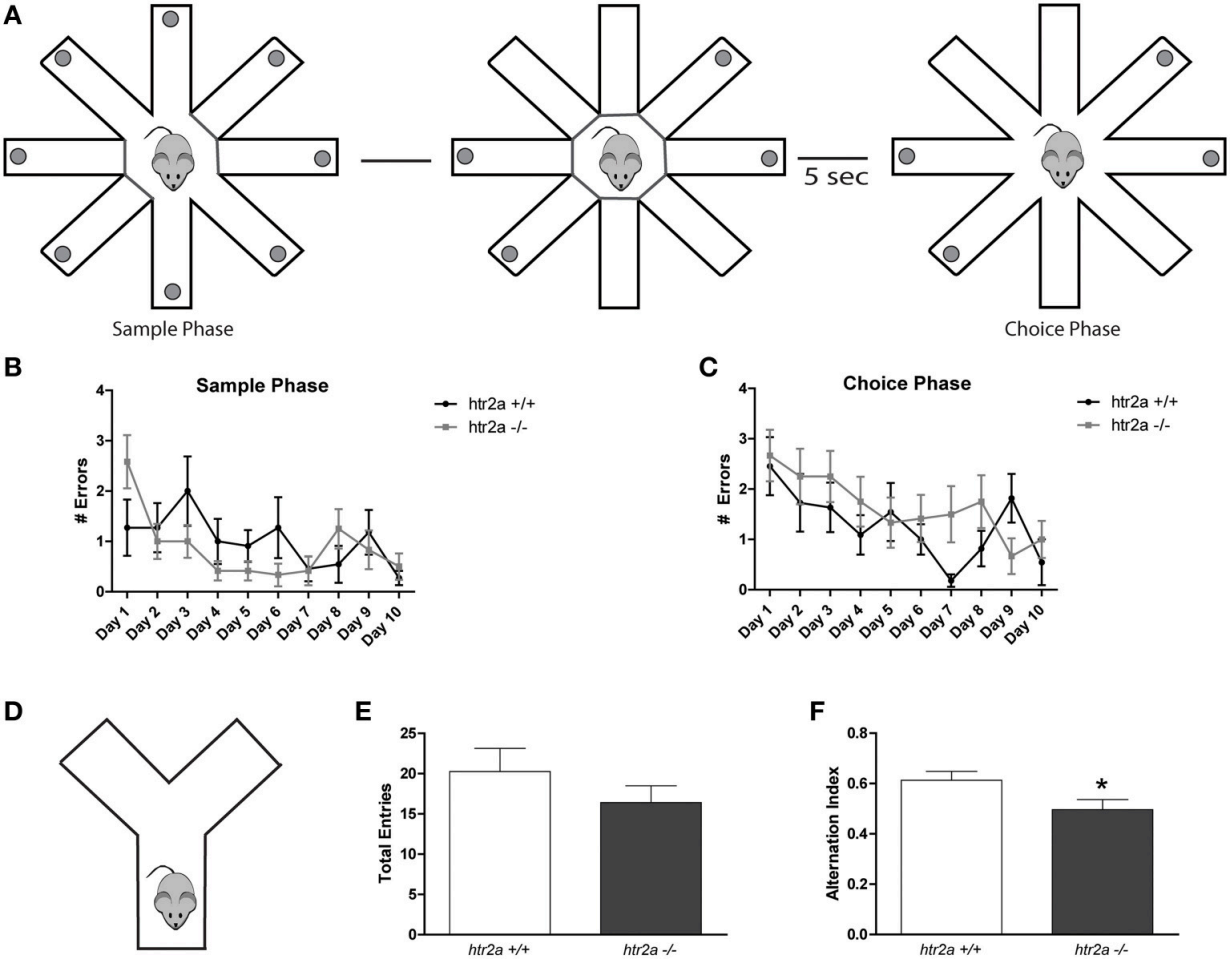
**Serotoninergic modulation via 5-HT2AR is required for the resolution of working memory tasks with high level of interference**. **(A)** Training and test scheme for the Radial Arm Maze test. Food-deprived mice were exposed to a single trial per day during 10 days. The delay between sample and choice phase was 5 s. **(B)** Number of errors made during sample phase. **(C)** Number of errors made during choice phase. *n* = 10 per group, *p* > 0.2, Two-way ANOVA. **(D)** Scheme of the Y-maze employed. Animals were located at the end of one of the tree arms and were allowed to explore the maze for 8 min. **(E)** Total number of entries to the Y-maze arms. **(F)** Alternation index made by the mice during the Y-maze spontaneous alternation test. *n* = 12 per group, ^*^p < 0.05, Student's *t*-test.

In order to evaluate if the deficit observed was due to a general effect of-HT2a signaling in mPFC function we tested *htr2a*^+/+^ and *htr2a*^−/−^ mice in two working memory tasks. The first one was the RAM maze (see Figure 4A). We used one trial per day and a fixed delay of 5 s between sample and test phase. In the sample phase animals were allowed to retrieve 4 food pellets from 4 of the 8 arms. In the choice phase all 8 arms were opened and visits to any of the arms opened during the sample phase were scored as working memory errors. We found that there were no significant differences between *htr2a*^+/+^ and *htr2a*^−/−^ mice in any of the phases of the experiments [see Figures 4B,C; *F*_genotype sample phase(1, 20)_ = 0.3910, *p* = 0.5385; *F*_genotype choice phase(1, 21)_ = 1.148, *p* = 0.296; *F*_errors ph1(9, 21)_ = 3.171, *p* = 0.0014; *F*_errors ph2(9, 21)_ = 3.341, *p* = 0.0008]. The second task was the spontaneous alternation Y-maze task (see Figure 4D). In this case, we found a deficit in alternation in *htr2a*^−/−^ compared with *htr2a*^+/+^ mice although they were no differences in the total number of entries performed during the task [Figure 4E; *t*_(22)_ = 1.076, *p* = 0.2936 and Figure 4F; *t*_(22)_ = 2.184, *p* = 0.0399]. During the RAM task, only one trial per day was used. The level of interference was thus very low between successive trials (separated by a 24 h delay). In contrast, the spontaneous alternation is a task but has a high level of interference since the animals were allowed to explore the maze as much as they wanted for 8 min without interruption. Our results thus suggest that the deficit observed in *htr2a*^−/−^ mice might not be due to a working memory problem *per se* but to a deficit in interference control.

### 5-HT1aR blockade rescues the deficit observed in the OIC task in *htr2a*^−/−^

**Figure 5:**
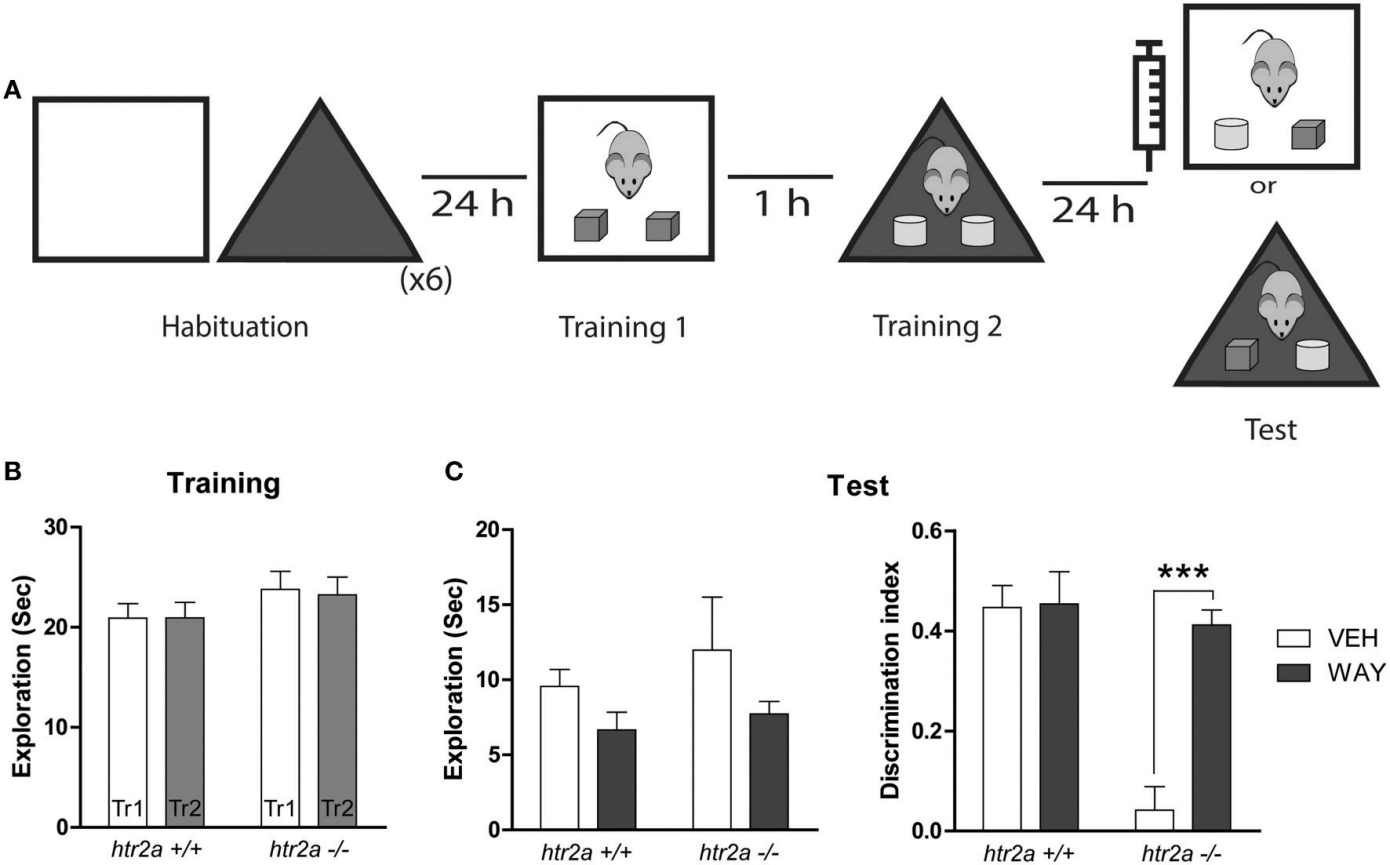
**Blockade of 5-HT1AR rescues the phenotype observed in the OIC task in ***htr2a***^**−/−**^ (A) Training and test scheme for the object-in-context (OIC) task**. Fifteen minutes before the test session mice were infused with 1 μg/side of WAY 100135 or vehicle (VEH) into the mPFC. **(B)** Exploration time measured in seconds made by the mice during the first and the second Training phases (Tr 1 and Tr2). **(C)** Total exploration measured in seconds (left) and Discrimination Index (right). Discrimination index was calculated as the time spent exploring the incongruent object minus the time spent exploring the congruent one over the total exploration time during the test session. *n* = 6 per group, ^***^p < 0.0001, Two-way repeated measured ANOVA followed by Bonferroni‘s *post-hoc* test.

The mPFC is highly enriched with 5-HT1aR and 5-HT2aR. Thus, it was interesting to explore whether both receptors played a role in the serotoninergic modulation of mPFC function during the resolution of the OIC task. In order to test this possibility we infused a 5-HT1a selective antagonist, WAY-100135, in the mPFC 15 min before the test session (see Figure 5A). As was described before, there was no differences between genotypes during the training phase (see Figure 5B). We found an effect of the drug on total exploratory time [see Figure 5C; *F*_(1, 12)_ = 0.1718, *p* = 0.047] for both genotypes consistent with the previously reported result that WAY-100135 affects locomotion in a dose dependent manner (Wedzony et al., 2000). Concerning the discrimination levels between the congruent and incongruent object we found an interaction Genotype x Treatment [*F*_(1, 29)_ = 14.44, *p* = 0.0011]. The results of *post-hoc* analyses showed that WAY-100135 had no effect in the discrimination between the congruent and incongruent objects in *htr2a*^+/+^ *mice* (see Figure 5C), but restores the ability to discriminate between the congruent and incongruent objects in the *htr2a*^−/−^ mice (see Figure 5C).

## Conclusions

In the current study the constitutive loss of 5-HT2aR produce deficits in particular class of recognition memory. The deficits were reserved to the OIC and TMOR task while the performance of *htr2a*^−/−^ mice were normal in the SNOR. The deficit observed in the OIC task was rescued by antagonizing the 5-HT1aR in the mPFC before the test session. While the SNOR task can be solve only by taking into account the characteristics of the objects, the OIC and TMOR tasks require the animals to remember an association between the objects and the context in which they have seen them or the objects and their relative position in time. This suggested that 5-HT2aR signaling might be necessary to control the expression of the relevant memory traces when complex representations must be used for successful retrieval. Results from the two working memory tasks suggest that 5-HT2aR signaling is helpful to performance when the interference load is high, like when two familiar objects from different experiences are presented, but does not play a role when this interference load is low. These results then support a role of 5-HT2aR in interference control, probably acting at the mPFC level.

Previously, we have shown that blockade of 5-HT2aR with MDL 11939 in mPFC of rats during the test phase of an OIC task impaired the resolution of this task (Bekinschtein et al., 2013). Here we show that *htr2a*^−/−^ mice recapitulate this phenotype suggesting that the constitutive absence of the receptor signaling does not generate compensatory mechanisms and that it affects a specific type of recognition memory.

Recognition memory involved the interaction of different structures including the hippocampus, perirhinal and prefrontal cortices. Our model does not allow us to identify directly which subpopulation is responsible for the deficits observed. However, some inference can be made based in the results obtained. The deficits were observed in tasks that cannot be solved by a single item strategy suggesting that 5-HT2a signaling is necessary for the ability to reduce memory interference. The RAM results together with results obtained using a Morris water maze (data not shown) indicate that *htr2a*^−/−^ mice have no deficits in spatial navigation indicating normal hippocampal function in *htr2a*^−/−^ mice. Neither the deficits could be explained by differences in locomotor activity since we had previously shown that *htr2a*^+/+^ and *htr2a*^−/−^ mice showed no significant differences in this measure in many different locomotors dependent tasks (Weisstaub et al., 2006). In addition, unimpaired performance of *htr2a*^−/−^ mice in the SNOR suggests that the functional integrity of the perirhinal cortex—a structure that is essential for item recognition—(Barker et al., 2007; Bartko et al., 2007) is also normal in our mice. Our studies also indicate that *htr2a*^−/−^ mice have no deficits in the acquisition phase of these tasks and that they are able to distinguish a novel and familiar objects. Even more, our results observed in the different tasks evaluating recognition memory support the hypothesis that the deficit observed in *htr2a*^−/−^ are due to the key role that the 5-HT2aR play in mPFC function.

The two tasks in which we did find deficits in *htr2a*^−/−^ mice were the TMOR and OIC tasks. To solve them, mice have to integrate and compare information obtained during the training sessions. In one case (TMOR), the important information is of a temporal nature since the animals have to recognize the relative recency of the object experience (Barker et al., 2007; Bekinschtein et al., 2013). In the OIC task, the relevant information comes from the association of the context with the objects. In this case, both objects are familiar as well as the context in which they are presented during the test phase. The difficulty arises from the fact that one of the objects is presented in a different context during the sample phase. During the test phase there is an “inconsistency” between one of the objects and the context in which it is presented. Behaviorally, the animals explore more the “incongruent” than the “congruent” object. Although we do not know how the system solves this problem, we hypothesized that during the test phase the mPFC controls the retrieval of the memory traces, selecting the more relevant one. It has been shown that mPFC is important for the resolution of this type of tasks. Barker et al. found that mPFC excitotoxic lesions affected performance in TMOR and in an object-in-place task during which the animals have to remember which object has been seen and where it was (Barker et al., 2007; Barker and Warburton, 2011; Chao et al., 2015; de Souza Silva et al., 2015). Then, it is possible that the deficit observed in *htr2a*^−/−^ mice results from a lack of 5-HT2aR signaling in the mPFC in a similar way to what was observed in our previous work with rats and in this way affects the ability of the mPFC to interact with other structures to solve the task.

Other experiments support this hypothesis. *htr2a*^−/−^ mice showed deficits in the Y-maze spontaneous alternation task, without showing deficits in the RAM task. Both tasks assess working memory, a function highly dependent on mPFC integrity (Baeg et al., 2003; Benchenane et al., 2010; Wei et al., 2015). An important difference between both tasks resides in the designed used to test working memory. Our RAM task has a high memory load, since the animals need to keep in memory a certain number of arms (four) that already visited during sample phase in order to get the maximum amount of reward possible during a subsequent test phase. However, it has a low interference load as only one trial per day is presented to the animal. In the case of the Y-maze, the animals are allowed to explore the arms as much as they like and in the order they want and is based on the natural tendency of the mice to alternate the visits. Since this task has no reward associated with any of the visits, and the animals are left in the maze for a considerable lapse of time, the task is prone to produce high levels of interference between the successive visits of the arms. Then, we have two working memory tasks that differed in the memory load and level of interference and in which *htr2a*^−/−^ mice respond differently. These differences indicate that *htr2a*^−/−^ mice do not have a mPFC deficit in general or a working memory deficit *per se*. Instead they show a deficit in cases in which the interference level is high, suggesting that serotonin signaling through 5-HT2aR is involved in interference resolution necessary in specific type of working memory tasks. This interference control might act through a top-down executive control over other areas involved in the resolution of the tasks (Goldman-Rakic, 1995; Petrides, 1995; Kesner and Churchwell, 2011; Griffin, 2015). The deficits observed in the Y-maze task in absence of 5-HT2aR signaling could be explained by an imbalance in the top-down control made by the mPFC in the same way as what we saw in the TMOR and OIC tasks.

Although it is clear that serotonin plays an important modulatory role on mPFC function, how and through which receptors serotonin exerts these effects is far from clear. One of the main problems is the specific and sophisticated pattern of expression that show each 5-HT receptors subtype. Two of the main serotonergic receptors in the mPFC are the 5-HT2aR and 5-HT1aR. These two receptors exert, in the mPFC, opposite effects on neuronal activity. Since the interplay between these two receptor types is a key factor in serotonin modulation of cortical function, we decided to evaluate if they 5-HT1aR was also involved in the regulation for OIC task. We hypothesized that if both receptors played antagonistic roles in mPFC function, then we might be able to restore the deficit observed in *htr2a*^−/−^ mice by manipulating 5-HT1aR activity. To do this, we infused our genetically modified mice with a selective 5-HT1aR antagonist directly into the mPFC. By combining genetic, pharmacologic and stereotaxic strategies we were able to show that mPFC 5-HT1aR are also involved in the resolution of the OIC task, and that during retrieval in the absence of 5-HT2aR signaling the main effect is through the activation of 5-HT1aR. 5-HT2aR is densely expressed in layer V of the cortex, both in excitatory and inhibitory neurons. Interestingly 60% of 5-HT2aR pyramidal cells also co-expressed 5-HT1aR. These cells showed a clear compartmentalization regarding the expression pattern of these two serotonin receptor subtypes. While 5-HT2aR are expressed predominantly in the basal part of the apical dendrite, 5-HT1aRs are expressed in the axon initial segment from where they exert an inhibitory role over the generation of action potentials (Puig and Gulledge, 2011; Celada et al., 2013a). This segregation has been postulated to be key in regulating neuronal excitability at a local level but will also have long range effects, since many of the pyramidal cells that express these receptors project to different structures, including the raphe nucleus (Celada et al., 2001, 2013b). The activation of 5-HT1aR hyperpolarizes pyramidal neurons whereas activation of 5-HT2aR results in neuronal depolarization, reduction of the afterhyperpolzarization and increase of excitatory postsynaptic currents and discharge rate (Celada et al., 2013b), then the response of the cortex to 5-HT stimulation can be inhibition, excitation or biphasic, *in vitro* as well as *in vivo* (Celada et al., 2001, 2013b; Avesar and Gulledge, 2012). The responses observed in the raphe are not only due to the differences in the modulation of projection cells from the mPFC but also to the activation of different receptors and cell types in the raphe itself (Celada et al., 2001, 2002, 2013a,b). Then the absence of 5-HT2aR probably affects not only the firing pattern of pyramidal cells in the mPFC (Weisstaub et al., 2006) but might also affect the response of the raphe nucleus to a particular stimulus. It is possible that the absence of 5-HT2aR, switch the balance to increase inhibition of projection cells, decreasing the stimulation received by the raphe and then affecting the release of 5-HT onto the cortex. If serotonin signaling in the cortex is important for retrieval control, then these changes could be, in part, responsible for the deficit observed in the *htr2a*^−/−^ mice. Although our model does not allow us to identify if the effects observed behaviorally are due to the activation of 5-HT1aR that are co-expressed with 5-HT2aR or the ones expressed in other cortical cells, our results indicate that both receptor types are involved. More specific manipulations might allow in the future determining which subpopulations of 5-HT1aRs as well as 5-HT2aRs are responsible for the effects observed.

We have shown that the ablation of 5-HT2aR signaling throughout development produces a deficit in recognition memory. This deficit appears to be selective to tasks that cannot be solved by single item strategy suggesting that 5-HT2aR signaling is involved in interference resolution. The normal performance of *htr2a*^−/−^ mice in the SNOR and RAM tasks support this hypothesis. In addition the phenotype observed in *htr2a*^−/−^ mice is consistent with the phenotype we observed in rats (Bekinschtein et al., 2013) suggesting that the constitutive absence of the receptor signaling does not generate compensatory mechanisms. The congruence between the results in both species implies that the main effect of 5-HT2aR signaling in the mPFC is during the retrieval phase of the memory process. In the absence of 5-HT2aR signaling, the behavioral effect observed appears to be due to the activation of 5-HT1aR receptors in the mPFC suggesting that serotonin modulation of mPFC function is a key element for recognition memory in rodents.

Frequently serotonin and its receptors are associated with psychiatric disorders. However, deficits in serotonin system appear to be involved in processes that is seen as the main characteristics of these disorders and that span across many of them. These deficits are more specific and selective, even in the complete absence of 5-HT2aR expression; there are no global memory deficits but rather particular features of the memory process that are affected. Then, there is a potential for members of the serotoninergic system to be use as a biological marker of cognitive processes in the normal brain.

In summary, this work support emerging evidence that serotonergic system in the mPFC is involved in memory retrieval. Since episodic memory is affected in pathologies such as schizophrenia, Alzheimer, frontotemporal dementia and depression. Our results point out to the 5-HT1a and 5-HT2a receptors as novel target for drug development to improve episodic memory retrieval in these psychiatric and neurological disorders.

## Author contributions

JM: Carried out the recognition memory experiments, analyzed the data and helped to wrote the manuscript. LC: Performed spontaneous alternation task. GM: Trained NW and supervised her for the RAM experiment, discussed the results and helped with the manuscript. JG: Provided the mice, discussed the results and helped with the manuscript. PB and NW conceived the project. PB discussed the results and helped with the manuscript. NW: Wrote the paper and supervised all aspects of the project.

## Funding

Research grants from National Agency of Scientific and Technological Promotion of Argentina (ANPCyT) to NW (PICT 2008-00072, PICT 2008-1065 and PICT 2012-0927).

### Conflict of interest statement

The authors declare that the research was conducted in the absence of any commercial or financial relationships that could be construed as a potential conflict of interest.
